# Acute effects of lactate infusion on metabolism, AD biomarkers, and cognition: The LEAN study

**DOI:** 10.1002/alz.70984

**Published:** 2025-12-10

**Authors:** Riley E. Kemna, Paul J. Kueck, Anneka Blankenship, Casey S. John, Chelsea N. Johnson, Zachary D. Green, Hana Mayfield, Lauren Yoksh, Lance Johnson, John P. Thyfault, Jonathan D. Mahnken, Benjamin F. Miller, Jill K. Morris

**Affiliations:** ^1^ University of Kansas Alzheimer's Disease Research Center Fairway Kansas USA; ^2^ Department of Neurology University of Kansas Medical Center Kansas City Kansas USA; ^3^ Department of Cell Biology and Physiology University of Kansas Medical Center Kansas City Kansas USA; ^4^ Department of Physiology University of Kentucky Lexington Kentucky USA; ^5^ Sanders Brown Center on Aging University of Kentucky Lexington Kentucky USA; ^6^ Internal Medicine‐Division of Endocrinology University of Kansas Medical Center Kansas City Kansas USA; ^7^ Diabetes Institute University of Kansas Medical Center Kansas City Kansas USA; ^8^ Department of Biostatistics & Data Science University of Kansas Medical Center Kansas City Kansas USA; ^9^ Aging and Metabolism Research Program Oklahoma Medical Research Foundation Oklahoma City Oklahoma USA; ^10^ Oklahoma City Veterans Association Oklahoma City Oklahoma USA

**Keywords:** aging, Alzheimer's disease, exercise, fluid biomarkers, lactate, metabolism

## Abstract

**BACKGROUND:**

Impaired cerebral glucose metabolism is a hallmark of Alzheimer's disease (AD). Lactate is an alternative brain fuel; however, whole‐body lactate metabolism has not been measured in AD.

**METHODS:**

The Lactate for Energy and Neurocognition Trial (NCT05207397) was a single‐arm trial that enrolled 24 cognitively healthy (CH) older adults and 12 cognitively impaired (CI) participants. Subjects underwent a stable isotope lactate infusion to evaluate lactate metabolism, cognitive testing, and blood biomarker analyses. pTau217, brain‐derived tau (BD‐tau), pTau181, glial fibrillary acidic protein (GFAP), neurofilament light chain (NfL), total tau, and brain‐derived neurotrophic factor (BDNF) were analyzed by Simoa HD‐X (Quanterix).

**RESULTS:**

Lactate metabolic clearance rate did not differ between CH and CI subjects (*p* = 0.988). After infusion, global cognition was improved (*p* < 0.001) and plasma pTau217 (−33.8%, *p* < 0.001), BD‐tau (−32.6%, *p* < 0.001), pTau181 (−21.4%, *p* < 0.001), GFAP (−39.7%, *p* < 0.001), and NfL (−19.5%, *p* < 0.001) were reduced.

**CONCLUSIONS:**

Lactate turnover was not different between diagnosis groups. Lactate infusion improved cognition and reduced AD fluid biomarkers.

**Highlights:**

- Individuals with Alzheimer's disease (AD) can metabolize lactate as well as healthy controls.
- Lactate infusion might improve global cognition and processing speed.
- Lactate infusion results in significant decrease of AD fluid biomarkers.

## 1 INTRODUCTION

Alzheimer's disease (AD) is the most prevalent form of dementia, affecting millions of older adults each year.^1^ Emerging therapies for AD are primarily focused on removal of β‐amyloid and show limited efficacy, with some slowing progression of cognitive decline, but none able to halt or reverse cognitive impairment.^2^ Because of these current limitations, alternative approaches to reduce disease risk are essential. Lifestyle factors, such as physical activity and exercise, impact brain health and could play an important role in reducing risk of cognitive decline.^3^ Exercise has been shown to increase hippocampal volume^4^ and improve cognitive function in healthy individuals.^5^ However, exercise benefit to cognition is variable,^6^, ^7^ and further investigation is needed to understand its nuanced effects. The underlying mechanisms are likely multifaceted, as exercise has been shown to improve cardiovascular health,^8^, ^9^ reduce inflammation,^10^, ^11^ and improve insulin sensitivity.^12^, ^13^ Transient benefits of acute bouts of exercise could also benefit brain health through increased release of growth factors that contribute to neurogenesis and neuroplasticity,^14^ or increased systemic production of lactate.

While lactate was once thought to be a waste product during exercise, it has become increasingly appreciated as a beneficial and necessary metabolite that is highly produced during exercise.^15^ Lactate is readily used by many tissues such as skeletal muscle, the heart, kidneys, and the brain,^16^, ^17^, ^18^, ^19^ and some studies have shown that it acts as a preferential fuel source for neurons.^20^, ^21^ In hypoglycemia‐induced cognitive impairment during a hyperinsulinemic–euglycemic clamp, lactate infusion can rescue cognition,^22^, ^23^ suggesting lactate as a sufficient alternative substrate that can be used by the brain. The potential role for lactate to satisfy metabolic need is particularly relevant in the case of AD, where whole‐body insulin sensitivity is impaired^24^ and cerebral hypometabolism of glucose is present.^25^, ^26^, ^27^ However, an individual's utilization of lactate as a fuel source is dependent on both lactate uptake and oxidation rates, which could explain why exercise benefits are variable. Furthermore, no work has been done to investigate how individuals with AD can use systemic lactate compared to cognitively healthy (CH) individuals.

To address this knowledge gap, we conducted the Lactate for Energy and Neurocognition (LEAN) clinical trial. LEAN was a single‐arm trial that enrolled CH and cognitively impaired (CI) participants and involved a “lactate clamp” (LC) procedure, during which lactate is infused with a goal of achieving a steady state of increased blood lactate concentrations. Stable isotopes were infused during the lactate clamp in order to measure whole‐body lactate metabolism. Our a priori hypothesis was that the CI group would have a lower average metabolic clearance rate (MCR) of lactate compared to CH and that lactate infusion would improve cognition in both CH and CI groups. We did not observe group differences in whole‐body lactate metabolism, suggesting individuals with CI can utilize lactate as a fuel source equally with CH individuals. Our secondary objective was aimed at assessing the acute effect of lactate on global cognition, where we observed improved global cognition specifically driven by improvements in processing speed. We further explored the acute response of AD‐relevant plasma biomarkers to lactate infusion and effects of lactate on markers of blood–brain barrier (BBB) permeability and kidney function.

## 2 METHODS

The LEAN study was designed to investigate potential differences in systemic lactate metabolism in individuals with and without AD. Detailed methodology for the LEAN study has been published.^28^ LEAN was approved by the Institutional Review Board (IRB) (Study #144303) at the University of Kansas Medical Center and is registered on clinicaltrials.gov (NCT05207397, registered January 26, 2022). This study complies with the Declaration of Helsinki and all participants provided informed consent. The primary outcome of the study was to determine lactate metabolic clearance rate in CH or CI older adults. The secondary outcome was to measure global changes in cognitive performance with elevated blood lactate levels. We performed an LC procedure, during which unlabeled lactate was infused until a blood lactate concentration of 4–5 mM was reached. The LC was accompanied by infusion of a stable isotope of lactate to determine lactate kinetics. All other reported measures were exploratory analyses performed to investigate how lactate metabolism might affect AD biomarkers.

### 2.1 Participants

Twenty‐four research participants were recruited through the University of Kansas Alzheimer's Disease Research Center (KU ADRC). The study enrolled participants from April 2023 through March 2024. Participants went through a telephone screening for inclusion/exclusion criteria by study staff. These methods have been previously described.^28^ Briefly, participants were evaluated using a remote Clinical Dementia Rating (CDR). The CDR scale is used to stage dementia clinically and uses a semi‐structured interview to evaluate six domains of cognition and function, which combine to compute a global CDR.^29^ Participants were subsequently diagnosed by the evaluating clinician as either cognitively healthy (*n* = 12) or cognitively impaired (*n* = 12). Eligible participants were 60 years or older, post‐menopausal, on stable medication doses of 1 month or greater, and were either cognitively normal or had cognitive impairment defined as a current diagnosis of AD. Exclusion criteria included diagnosis of a clinically significant chronic disease such as cardiovascular disease (CVD), metabolic diseases, cancer, human immunodeficiency virus (HIV), chronic kidney disease (CKD), or acquired immunodeficiency syndrome. Participants were also excluded if they were unable to provide consent, had type 1 diabetes, or venous access could not be obtained.

**RESEARCH IN CONTEXT**

1. **Systematic review**: A literature review (PubMed) was conducted for relevant research related to whole‐body lactate metabolism in Alzheimer's disease (AD), impact of lactate on cognition, and acute response to lactate infusion. Little work has been done to understand how lactate metabolism is affected in AD or how it might affect cognition.
2. **Interpretation**: Our findings demonstrate that individuals with AD do not have impaired whole‐body lactate metabolism compared to healthy controls, and that lactate could acutely improve cognition and reduce AD fluid biomarkers.
3. **Future directions**: Future work is needed to understand mechanisms that underly improvements in cognition and reduced AD fluid biomarkers following lactate infusion.

### 2.2 Study flow

The LEAN study was performed in one visit to the Clinical and Translational Science Unit (CTSU) at the University of Kansas Medical Center. This was a single‐arm study focused on characterizing lactate turnover and determining the effect of lactate on cognitive performance. Participants arrived at the CTSU following an overnight fast. Information regarding medication usage and medical history was gathered, as well as height and weight. Participants underwent a dual‐energy x‐ray absorptiometry (DXA) scan to assess body composition. Two intravenous catheters were placed, with one in each arm, by a trained nurse, and participants rested approximately 10 min prior to completing cognitive testing (NIH Toolbox). The hand of the non‐infusion arm, from which blood specimens are collected, was placed in a heated hand box set at 55°C for arterialization of venous blood. Baseline sampling of blood and breath was collected after cognitive testing. Tracer boluses were administered prior to beginning stable isotope and unlabeled infusions, which lasted for 120 min. Breath and blood specimens were collected at designated timepoints throughout the infusion. The second cognitive test battery was performed at 90 min of lactate clamp during steady‐state conditions of the infusion. All study visits were performed at the same time in the morning to reduce any diurnal variation in metabolism and AD biomarkers.^30^

### 2.3 DXA scan

Participants were asked to void prior to receiving a DXA scan to measure body composition (GE Lunar iDXA). Data collected included fat mass, fat‐free mass, bone mineral density, and body fat percentage.

### 2.4 LC and tracer infusions

The LC procedure and tracer infusion have been previously described.^28^ Following baseline sample collection and cognitive testing, priming boluses of 57.5 mg [^13^C_3_] lactate, 250 mg [6,6‐H^2^] glucose (D_2_‐glucose), and 136 mg H^13^CO_3‐_ were administered. Boluses were immediately followed by continuous infusions of [^13^C_3_] lactate at a rate of 5 mg/min and D_2_‐glucose at a rate of 2 mg/min, rates that were held constant during the clamp for all subjects. Unlabeled lactate infusion was started at a rate of 2.6 mg/kg*min. At each timepoint of sample collection, fresh whole blood collected in ethylenediaminetetraacetic acid (EDTA) anticoagulant was tested by a handheld lactate meter (Nova Biomedical), and the unlabeled sodium lactate infusion rate was adjusted appropriately to obtain a goal blood–lactate concentration of 4–5 mM, which is similar to blood lactate during moderate‐intensity exercise.^14^, ^31^

### 2.5 Cognitive assessment

Participants underwent cognitive tests (NIH Toolbox) at baseline and 90 min of the LC. After IV placement, participants rested for 10 min, and then a baseline test was administered. A second test was administered at 90 min, after blood lactate reached the target concentration of approximately 4 mM. The NIH Toolbox battery included the Flanker Inhibitory Control and Attention Test, Picture Sequence Memory Test, and Pattern Comparison Processing Speed Test. This test battery has been previously described^32^ and separate versions of tests were administered pre and post infusion when available (Picture Sequence tests). Cognitive scores were normalized for age, sex, and education and are presented as *t*‐scores or National Percentiles. Global cognition was calculated as a composite of all three cognitive domain *t*‐scores.

### 2.6 Breath collection

Breath sampling was performed at baseline, 60, 75, 90, and 120 min. Breath samples were collected in 10 mL glass tubes (#139B/NP). Samples were analyzed by isotope ratio mass spectrometry (IRMS) for ^13^CO_2_ by Metabolic Solutions (Nashua, NH).

### 2.7 Blood collection and processing

Blood samples were collected at baseline and at 10, 20, 30, 45, 60, 75, 90, and 120 min. Specific blood volumes are detailed in our previously published manuscript.^28^ Blood was collected in tubes with acid citrate dextrose (ACD) anticoagulant for apolipoprotein E4 *(APOE4)* genotyping, EDTA anticoagulant for biomarker analyses, and potassium oxalate/sodium fluoride for isotopic enrichment.

*APOE* genotype was determined by TaqMan single nucleotide polymorphism allelic discrimination assay (Thermo Fisher). Participants were classified as an *APOE4* carrier if they carried at least one *APOE4* allele. From blood collected in EDTA, we isolated platelet‐rich plasma (PRP) by centrifugation at 1500× g for 10 min at 4°C. We then isolated platelet‐poor plasma (PPP) by a second centrifugation at 1700× g for 15 min at 4°C. PPP was collected at baseline, 45, 60, 75, and 120 min. PRP and PPP were stored at −80°C for later analyses. In potassium oxalate/sodium fluoride tubes, they were collected at baseline, 60, 75, 90, and 120 min. Following collection, whole blood and 8% perchloric acid were mixed 1:1 to hemolyze blood cells for release of lactate. Samples were incubated at 4°C for 5 min, followed by centrifugation at 1500× g for 10 min. The supernatant was collected and centrifuged at 1700 × g for 15 min. The supernatant was collected and stored at −80°C for determination of isotope enrichment and concentration.

### 2.8 Isotope analyses

Lactate and glucose were prepared using our previously published methods^31^, ^33^, ^34^ for gas chromatography–mass spectrometry (GC‐MS) using the heptafluorobutyric anhydride and pentaacetate derivatives, respectively. To start, an exchange column of anion resin (AG 1‐X8, 100–200 mesh formate form) was washed with 10 mL 2 M formic acid followed by two washes of 10 mL water. A second exchange column of cation resin (AG 50‐X8, 100–200 mesh hydrogen form) was then placed on the anion resin column. Both columns were washed twice with water. Samples were neutralized with 2 M KOH then transferred to the ion exchange columns. Using water, glucose was eluted and collected first. Lactate was then eluted and collected separately using 2 M formic acid. Both eluted glucose and lactate were dried under vacuum. Dried glucose was reconstituted with methanol, transferred to a glass GC vial, dried under a stream of N2 gas, reacted with 100 µL of 2:1 acetic acid:pyrimidine for 1 h at 100°C, dried under a stream N2 gas, then reconstituted in ethyl acetate for GC‐MS analysis. Dried lactate was reacted with 200 µL 2,2‐dimethoxypropane and 10 uL 10% HCl in methanol for 1 h at room temperature. Next, 50 µL *N*‐propylamine was added and heated at 100°C for 1 h. The entirety was transferred to GC vials, dried under gas stream of N2, then reacted with 20 µL heptafluorobutyric anhydride at room temperature for 5 min. Vials were dried under a stream of N2 gas, then reconstituted with ethyl acetate for GC/MS analysis.

Lactate was analyzed on an 8890GC coupled to a 7090BMS in PCI mode on an Agilent DB‐17 column. Helium was used as the carrier gas and methane was used as the reactant gas. Inlet temperature was 250°C. Oven was initially held for 3 min at 80°C then ramped 35°C/min to 255°C. Ions monitored were 328 and 331 for the m+0 and the m+3 tracer, respectively. Glucose was analyzed on a 7890GC coupled to a 5977BMS in PCI mode on an Agilent DB‐5MS column. Helium was used as the carrier gas and methane was used as the reactant gas. Inlet temperature was 250°C. Oven was initially held for 1 min at 100°C then ramped 20°C/min to 300°C. Ions monitored were 331 and 333, for the m+0 and the m+2 tracer, respectively.

### 2.9 Calculations

Rate of appearance (*R*_a_), rate of disappearance (*R*_d_), and metabolic clearance rate for lactate and glucose were calculated using the modified Steele equation as described previously:^31^, ^33^, ^34^

1. *R*_a_ (mg*kg^−1^ *min^−1^) = {*F* – *V* [(*C*_1_+*C*_2_)/2] × [(IE_2_ – IE_1_)/(*t*_2_ – *t*_1_)]}/[(IE_2_ – IE_1_)/2]
2. *R*_d_ (mg*kg^−1^ *min^−1^) = *R*_a_ – [V (*C*_2_ – *C*_1_)/(*t*_2_ – *t*_1_)
3. MCR (mL*kg^−1^*min^−1^) = *R*_d_/[(*C*_1_ + *C*_2_)/2]

where *F* is the isotope infusion rate (mg/kg/min), *V* is the volume distribution of lactate and glucose and is equal to plasma volume (180 mL/kg); *C*_1_ and *C*_2_ are concentrations at sampling times *t*_1_ and *t*_2_; IE_1_ and IE_2_ are the excess isotopic enrichments of glucose and lactate. Measured IE values were corrected for background of blood samples taken before isotope infusion.

Lactate rate of oxidation (*R*_ox_) was calculated from measures of expired CO_2_ and IE_CO2_:

1. Relative lactate oxidation (%) = (IE_CO2_ *V*_CO2_ × 100)/*Fk*
2. Lactate *R*_ox_ (mg*kg^−1^ *min^−1^) = *R*_d_ × relative lactate oxidation

where IE_CO2_ is the isotopic enrichment of expired ^13^CO_2_, *V*_CO2_ is the volume of expired CO_2_ per minute, *F* is the [^13^C_3_] lactate infusion rate, and *k* is the correction factor for the retention of CO_2_, assumed to be 66.67 as determined by previous work in young, healthy male subjects.^31^

### 2.10 Plasma biomarker analyses

EDTA plasma was analyzed for pTau217, pTau181, BD‐Tau, NfL, GFAP, total tau, and brain‐derived neurotrophic factor (BDNF) using the Simoa HD‐X (Quanterix, Billerica, MA) according to manufacturer instructions. PRP was used for pTau217, pTau181, BD‐Tau, NfL, GFAP, and total tau measures. PPP was used for BDNF measures. Amyloid‐β 42 and 40 were assessed in EDTA plasma using a Lumipulse G1200 (Fujirebio, Tokyo, Japan). EDTA plasma was measured using enzyme‐linked immunosorbent assay (ELISA) for Claudin 5 (Biotechne, NBP2‐75332), Matrix metalloproteinase‐9 (MMP9; Invitrogen, BMS2016‐2), and Creatinine (RayBiotech, MA‐CTNSP‐2) according to manufacturer instructions. Hematocrit was measured from EDTA whole blood at each timepoint (Immunostics, Inc., Eatontown, NJ). EDTA plasma was also measured for lactate (ab65331) and glucose (Sigma) using colorimetric assays.

### 2.11 Estimated glomerular filtration rate

The estimated glomerular filtration rate (eGFR) was calculated using plasma creatinine according to the 2021 CKD‐EPI Creatinine Equation:

eGFR = 142 × min(SCr/*κ*,1)*^α^* × max(SCr/*κ*,1)^−1.200^ × 0.9938^Age^ × 1.012 [if female]

 where eGFR is mL/min/1.73 m^2^, SCr is creatinine (mg/dL), *κ* = 0.7 (females) or 0.9 (males), *α* = −0.241 (females) or −0.302 (males), min (minimum of SCr/*κ* or 1), max (maximum of SCr/*κ* or 1), and age (years).^35^

### 2.12 Estimated plasma volume

Estimated plasma volume (PV) was calculated using hematocrit and total blood volume (TBV) estimated by weight.^36^, ^37^ PV percent change was then calculated between baseline and 120 min PV.

1. TBV = weight (kg) × 70 mL/kg for male or weight (kg) × 65 mL/kg for female
2. PV = TBV (1 – hematocrit).

### 2.13 Statistical analysis

Our primary outcome measure was MCR and whether this differed between CH and CI individuals. Group means for lactate and glucose MCR, *R*_a_, and *R*_d_ were compared by two‐sample, unpaired *t*‐tests. We also used this test to compare endogenous *R*_a_, percent lactate oxidized, and lactate oxidation rate.

Our secondary outcome measure was change in global cognition in the overall group in response to lactate infusion. We first compared cognitive test differences between baseline and 90 min of the LC across the entire cohort by paired *t*‐tests and then characterized changes within groups. Baseline cognitive tests were compared between CH and CI groups using two‐sample, unpaired *t*‐tests. Categorical measures were tested by Pearson chi‐square test. To address potential concerns about differences in age between CH and CI groups, a linear model was constructed for lactate MCR adjusting for cognitive status, age, sex, and the interaction between age and cognitive status. Linear contrasts were then evaluated for overlapping ages (67.5, 70, 72.5, and 75 years old) to conduct these age‐ and sex‐adjusted between‐group comparisons. Residual histograms, residual versus predicted, and Normal Quantile‐Quantile plots were assessed to determine model fit and corresponding nonparametric tests (e.g., Wilcoxon rank sum test) were performed when indicated.

Exploratory analyses to assess biomarker response to the LC were performed on measures of pTau181, BD‐Tau, NfL, GFAP, total tau, BDNF, Claudin 5, MMP9, and creatinine at baseline and 120 min by paired *t*‐test. Percent change was calculated for each biomarker and analyzed by unpaired *t*‐tests to assess any potential differences between diagnosis groups.

Statistical analyses were performed in SPSS 29.0 (IBM) and SAS 9.4, and figures were made in GraphPad (Prism).

Sample size. No studies have assessed MCR in CH older adults or CI individuals. Because individuals with AD often exhibit insulin resistance, we have based our sample size estimate for the LEAN study on prior work in insulin‐resistant individuals showing an effect size of 1.4.^38^

## 3 RESULTS

### 3.1 Cohort demographics

Our study cohort consisted of 24 older adults that were either cognitively healthy (*n* = 12) or CI (*n* = 12). Study flow is described in the CONSORT diagram (Figure S1). CH and CI groups did not differ by sex, number of *APOE4* carriers, body mass index (BMI), lean mass, resting glucose, or HbA1c (Table 1). Our cohort included three individuals who identified as African American and one individual who identified as Native Hawaiian or other Pacific Islander. The CI group was significantly older with a mean age of 78.9 years, while the CH group had a mean age of 67.6 years (*p* < 0.001).

**TABLE 1:** Cohort demographics and baseline assessments.

Outcome measure	CH (*n* = 12)	CI (*n* = 12)	*p*‐Value
Age (years)	67.6 (5.4)	78.9 (7.6)	**<0.001** ^*^
Sex (% male)	7 (58.3%)	6 (50%)	0.682
CDR (0/0.5/1)	12/0/0	0/11/1	**<0.001** ^*^
Diagnosis (CH/MCI/AD)	12/0/0	0/8/4	**<0.001** ^*^
*APOE4* status (% carrier)	6 (50%)	7 (58%)	0.682
Body mass index	26.3 (5.5)	24.5 (3.5)	0.346
Lean mass (kg)	50.3 (12.8)	46.6 (8.3)	0.417
Glucose (mg/dL)	78.3 (19.9)	77.6 (11.4)	0.317
HbA1c	5.67 (0.4)	5.67 (0.3)	0.399
Aβ 42/40	0.095 (0.01)	0.076 (0.01)	**<0.001** ^*^
pTau217 (pg/mL)	0.28 (0.1)	1.17 (0.7)	**<0.001** ^*^
pTau181 (pg/mL)	23.0 (5.2)	44.4 (12.4)	**<0.001** ^*^
BD‐Tau (pg/mL)	6.6 (1.2)	11.0 (4.6)	**0.004** ^*^
NfL (pg/mL)	14.4 (3.1)	25.0 (9.1)	**<0.001** ^*^
GFAP (pg/mL)	128.8 (35.3)	277.0 (126.2)	**<0.001** ^*^
BDNF (pg/mL)	21.1 (16.2)	29.0 (24.4)	0.360
Total Tau (pg/mL)	0.731 (0.3)	1.05 (0.8)	0.211
Estimated glomerular filtration rate (mL/min/1.73 m^2^)	45.95 (12.7)	48.0 (13.4)	0.706
Flanker task (fully corrected *t*‐score)	46.7 (4.9)	39.4 (8.7)	**0.020** ^*^
Picture recall (fully corrected *t*‐score)	50.2 (9.6)	43.5 (6.9)	0.061
Processing speed (fully corrected *t*‐score)	51.33 (12.4)	39.5 (13.7)	**0.037** ^*^
Global cognition	49.4 (6.0)	40.8 (8.4)	**0.008** ^*^

*Note*: Characterization of participant demographics, body composition, baseline plasma biomarkers, and baseline cognitive scores. Values are given as means (SD). Measures that differ significantly between CH and CI groups are indicated in bold.

Abbreviations: AD, Alzheimer's disease; APOE4, apolipoprotein epsilon 4; BD‐Tau, brain derived tau; BDNF, brain‐derived neurotrophic factor; CDR, Clinical Dementia Rating (Global); CH, cognitively healthy; CI, cognitively impaired; GFAP, glial fibrillary acidic protein; MCI, mild cognitive impairment; NfL, neurofilament light; pTau, phosphorylated tau.

^*^ *p* < 0.05.

### 3.2 Baseline biomarker and cognitive assessments

We measured multiple biomarkers related to AD to characterize our study cohort. At baseline, our CI group had a lower Aβ 42/40 ratio compared to CH (*p* < 0.001). The CI group had higher measures of pTau217 (*p* < 0.001), pTau181 (*p* < 0.001), BD‐Tau (*p* < 0.001), NfL (*p* < 0.001), and GFAP (pg/mL, *p* < 0.001) (Table 1). CH and CI groups did not differ in levels of BDNF (pg/mL, *p* = 0.360) and total tau (pg/mL, 0.211) (Table 1).

### 3.3 Whole body lactate metabolism is not impaired in AD

**FIGURE 1:**
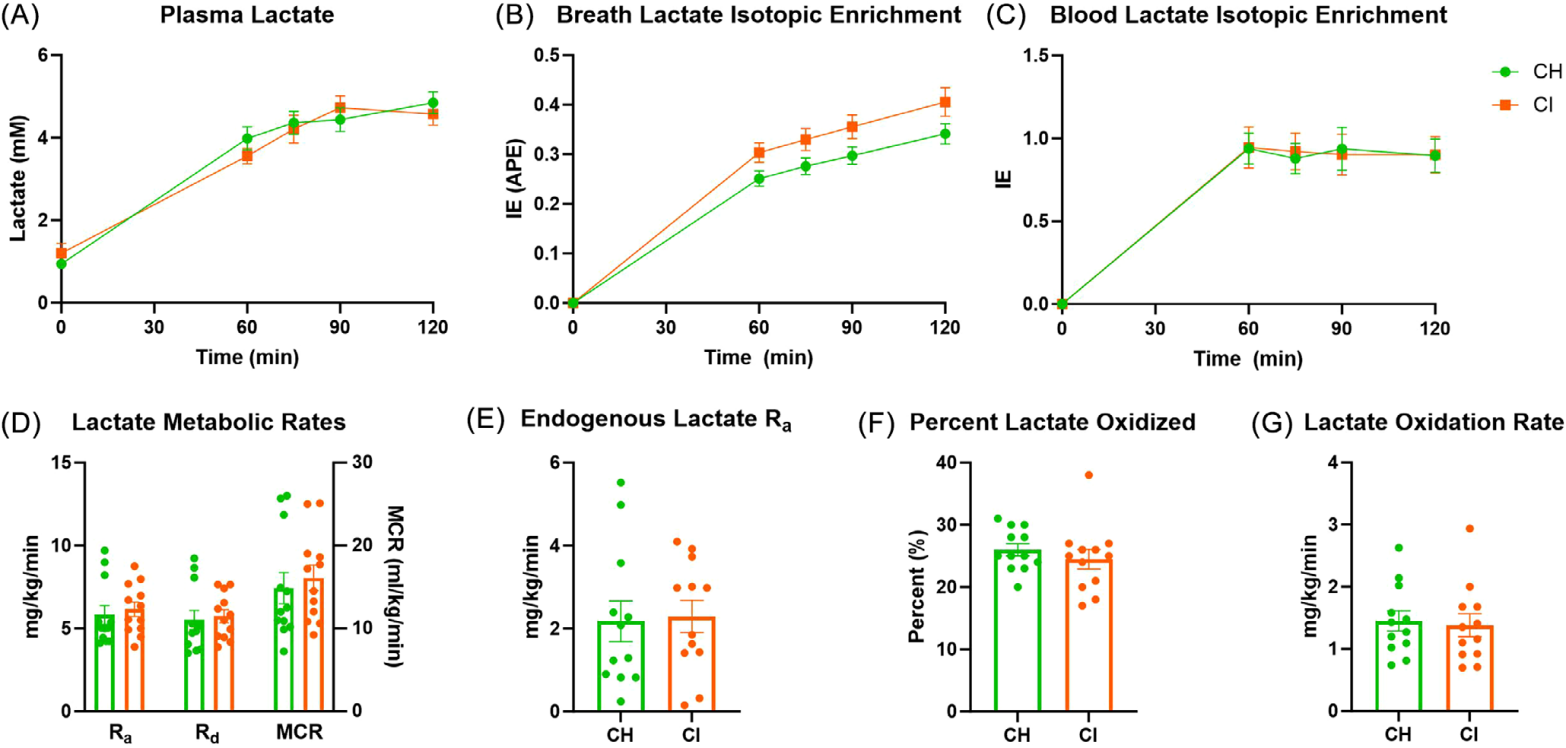
Lactate kinetics in individuals with cognitive impairment. (A) Plasma lactate concentrations throughout the lactate clamp. (B) Breath lactate isotopic enrichment. (C) Blood lactate isotopic enrichment throughout the lactate clamp. (D) Lactate *R*_a_, *R*_d_, and MCR. (E–G) Endogenous lactate *R*_a_ (E), percent of lactate oxidized (F), and rate of oxidation (G). CH, cognitively healthy; CI, cognitive impairment; MCR, metabolic clearance rate; *R*_a_, rate of appearance; *R*_d_, rate of disappearance.

**FIGURE 2:**
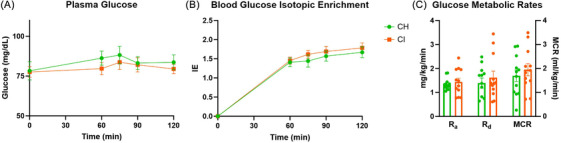
Glucose kinetics in individuals with cognitive impairment. (A) Glucose concentrations throughout the lactate clamp. (B) Blood glucose isotopic enrichment. (C) Glucose *R*_a_, *R*_d_, and MCR. CH, cognitively healthy; CI, cognitive impairment; MCR, metabolic clearance rate; *R*_a_, rate of appearance; *R*_d_, rate of disappearance.

Study participants underwent an LC procedure to determine potential differences in whole‐body lactate metabolism. Blood lactate concentrations were elevated during the LC compared to resting levels pre‐LC (Figure 1A) and did not differ by diagnosis group. Both CH and CI groups achieved target blood lactate concentrations of ∼4–5 mM, with average concentrations of 4.55 and 4.45 mM, respectively. Blood lactate held at the target concentration was safe and well tolerated in our cohort of older adults. Breath and blood isotopic enrichment of lactate approximated a steady state after 60 min of infusion (Figure 1B,C), as expected. We did not observe group differences between CH and CI measures for rate of appearance (*R*_a_), rate of disappearance (*R*_d_), or MCR for lactate (Figure 1D). After adjustment for age and sex, we did not find a significant difference in lactate MCR (120 min, baseline). Age, sex, and the interaction between age and cognitive status were not found to have a significant effect on lactate MCR (*p* > 0.5277). At overlapping ages, change in lactate MCR was higher for CI than for CH, but the change was not significantly different between groups (*p* > 0.4355). Furthermore, endogenous *R*_a_, percent lactate oxidized, and lactate *R*_ox_ did not differ between CH and CI (Figure 1E–G). Blood glucose concentration did not change between baseline and during LC (Figure 2A) and blood isotopic enrichment of glucose approximated a steady state after 60 min of infusion (Figure 2B). Glucose *R*_a_, *R*_d_, and MCR did not differ between CI and CH (Figure 2C).

### 3.4 Processing speed and global cognition are improved after lactate infusion

**FIGURE 3:**
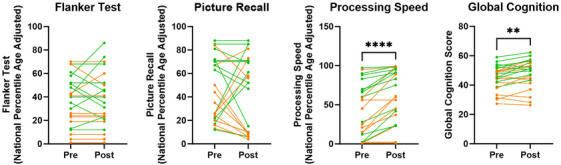
Cognitive test performance before and during steady state of lactate infusion. Cognitive performance was measured by NIH toolbox at baseline and 90 min of the LC to assess impact of lactate infusion on cognition. *p < 0.05. LC, lactate clamp; NIH, National Institutes of Health.

Cognitive assessments were conducted at baseline, with a second test performed at 90 min of the LC, when blood lactate had reached the steady‐state target concentration. As expected, CI individuals had lower cognitive scores in the Flanker task (*p* = 0.020), processing speed (*p* = 0.037), and global cognition (*p* = 0.008) compared to CH participants at baseline (Table 1). The CI group also trended toward reduced picture recall (*p* = 0.061) (Table 1). We did not observe significant changes in performance on the Flanker task (*p* = 0.728) and picture recall (*p* = 0.635) at 90 min of the lactate clamp compared to baseline. However, we did observe improved processing speed (*p* < 0.001) and global cognition (*p* = 0.001) within the whole cohort at this timepoint (Figure 3). When tested within diagnosis groups, processing speed was improved in both CH and CI groups, whereas global cognition was improved in the CH group (*p* = 0.003) and trended toward improvements in the CI group (*p* = 0.108) (Figure S2).

### 3.5 AD‐related biomarkers are decreased after lactate infusion

**FIGURE 4:**
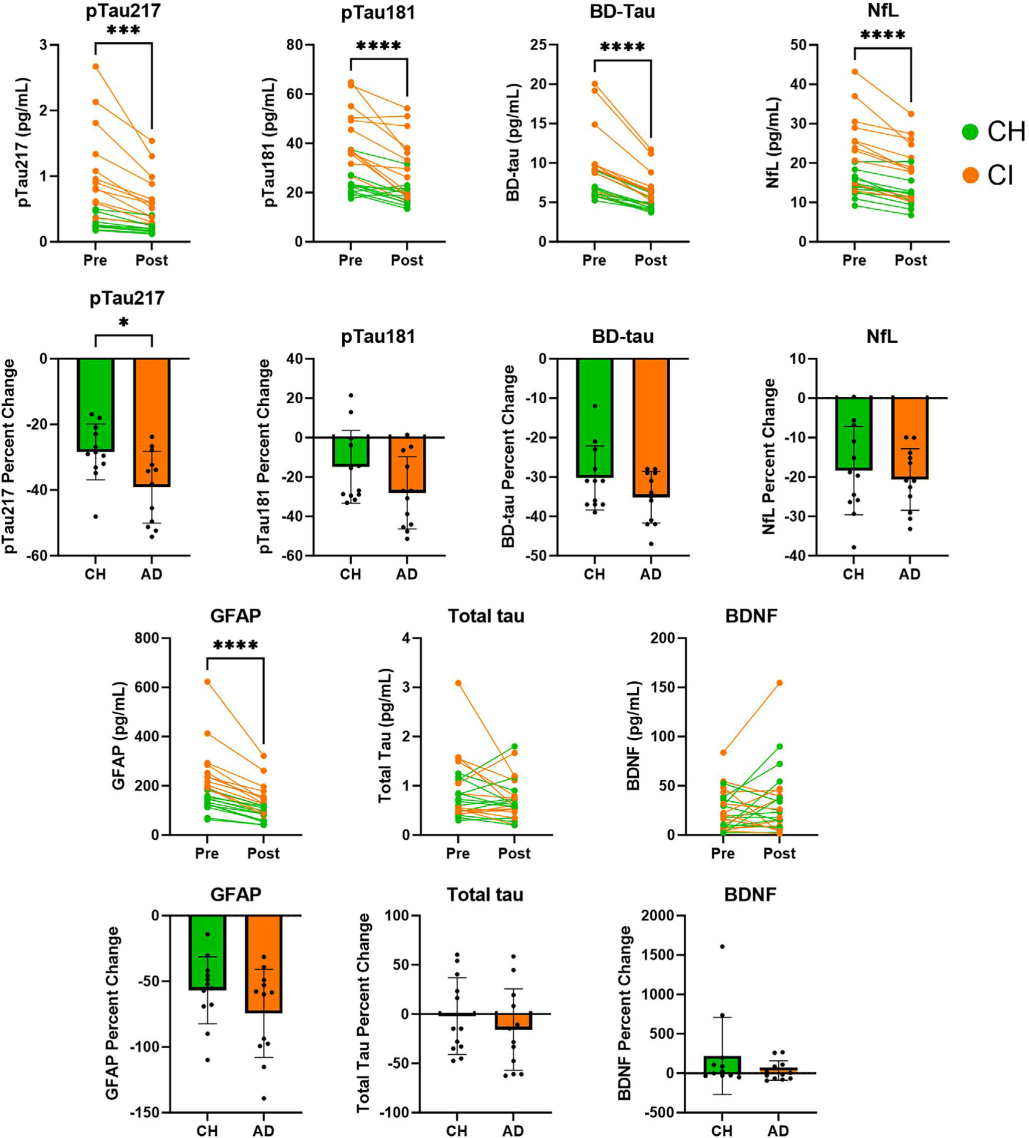
Acute AD plasma biomarker response to lactate infusion. Baseline and 120 min measures are presented for pTau217, pTau181, BD‐tau, NfL, GFAP, total tau, and BDNF, with percent change of each plasma biomarker below (C–H). AD, Alzheimer's disease; BD‐tau, brain‐derived tau; BDNF, brain‐derived neurotrophic factor; CH, cognitively healthy; CI, cognitive impairment; GFAP, glial fibrillary acidic protein; NfL, neurofilament light chain; pTau, phosphorylated tau. *p < 0.05.

To characterize the acute effects of increased blood lactate, we measured plasma biomarkers of AD. Strikingly, hallmark biomarkers of AD and neurodegeneration, including pTau217 (−33.8%, *p* < 0.001), pTau181 (−21.4%, *p* < 0.001), BD‐tau (−32.6%, *p* < 0.001), NfL (−19.5%, *p* < 0.001), and GFAP (−39.7%, *p* < 0.001), were all significantly decreased after the LC at 120 min compared to baseline levels. Other plasma biomarkers, including total tau and BDNF, were unchanged after the LC (Figure 4). A significant difference was found in the percent decrease in pTau217 (*p* = 0.0135) between cognitive groups, and the difference in pTau181 between groups approached significance (*p* = 0.0931). pTau217 percent change and pTau181 percent change were positively correlated (*p* < 0.001, *R* ^2^ = 0.614). To further measure biomarker response to lactate infusion, we measured pTau217 at 0, 10, 45, 90, and 120 min to investigate temporal changes in biomarker concentrations (Figure S3). At each time point, the CI group progressively decreased in plasma pTau217 with group mean (standard deviation [SD]) measures of 1.10 (0.51), 0.894 (0.38), 0.771 (0.35), 0.632 (0.30), and 0.681 (0.37) pg/mL, respectively. The CH group progressively decreased with group mean (SD) measures of 0.290 (0.11), 0.232 (0.10), 0.213 (0.10), 0.181 (0.083), and 0.205 (0.084) pg/mL.

### 3.6 BBB permeability is likely unchanged by lactate infusion

**FIGURE 5:**
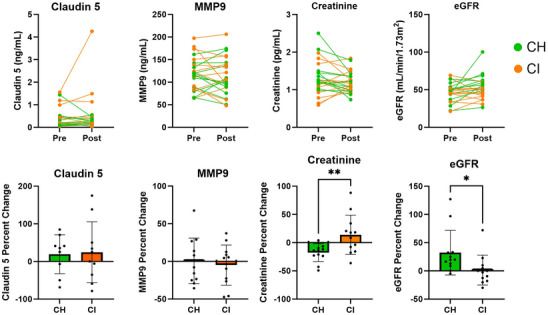
Biomarkers of blood–brain barrier permeability and kidney function were not changed after lactate infusion. Plasma Claudin 5, MMP9, creatinine, and eGFR at baseline and 120 min above. Percent change of Claudin 5, MMP9, creatinine, and eGFR below. CH, cognitively healthy; CI, cognitive impairment; eGFR, estimate of glomerular filtration rate; MMP9, matrix metalloproteinase‐9. *p < 0.05.

To investigate potential mechanisms that might explain the profound changes in AD biomarkers, we assessed plasma biomarkers of BBB permeability. Claudin 5, an endothelial cell tight junction protein that is considered a marker of BBB integrity, was not affected by the LC (*p* = 0.531). MMP9, another common plasma measure of BBB permeability, was not changed after the LC (*p* = 0.435) (Figure 5).

### 3.7 Kidney function did not change after LC

To further explore mechanisms underlying AD biomarker changes, we measured plasma creatinine in order to calculate an eGFR. Baseline eGFR was not different between groups (Table 1). Neither plasma creatinine nor eGFR was significantly different between subjects after the LC (Figure 5). However, CH had an increase in eGFR compared to CI, which was driven by a decrease in creatinine after the LC in the CH group. Changes in eGFR and creatinine were not related to observed changes in AD biomarkers. Blood volume did not differ significantly after lactate infusion (Figure S4) with a modest 4% increase, and was unrelated to any AD biomarker changes (Figure S4). Plasma volume, hematocrit, and hemoglobin values at each timepoint are reported in Table S1.

#### 3.7.1 Adverse events

There were no adverse events or serious adverse events in the LEAN trial.

## 4 DISCUSSION

Some studies indicate that exercise trials benefit cognitive outcomes in cognitively healthy older adults and those with AD, but not all studies are consistent. One key exerkine released during acute exercise is lactate. Lactate can freely cross the BBB and is postulated to serve as an alternative fuel source to glucose in the brain. However, whether whole‐body lactate metabolism (which may affect its availability in blood supplied to the brain) differs in aging and AD has never been tested. Thus, the primary goal of the LEAN trial was to investigate potential differences in whole body‐lactate metabolism in CI, with a secondary outcome to investigate potential effects of a lactate infusion on cognitive performance. Understanding how alternative fuel sources such as lactate are used in CI is critical, given that impaired cerebral glucose metabolism is a commonly observed hallmark of the disease.^25^, ^26^, ^27^

We observed that CH and CI groups had similar measures of *R*_a_, *R*_d_, and MCR of lactate and did not differ in lactate oxidation rate, percent lactate oxidized, and endogenous lactate *R*_a_. Because there was no difference in whole‐body lactate metabolism, this suggests that the systemic lactate clearance during exercise may be similar between CH and CI individuals. As lactate is BBB permeable and enters readily into the brain during both rest and exercise,^17^, ^18^, ^19^, ^39^, ^40^, ^41^ this suggests that both CH and CI individuals could stand to benefit equally from exercise‐induced increases in lactate. This is particularly important to understand in cases of decreased cerebral glucose metabolism, as is seen in AD. However, these studies were performed in a resting state, and exercise induced increases in fuel metabolism may change lactate utilization rates differently between participants with CH and CI and thus still need to be tested directly. While these measures were not different between groups, we did see heterogeneity within groups, which suggests some variability within our study population. This variability could be due to factors such as physical fitness, as exercise training can improve lactate clearance.^42^

Our secondary objective was to assess the effect of increased blood lactate on cognitive performance. Participants performed cognitive assessments before the lactate clamp and at 90 min, when blood lactate was at the target concentration and was held at a steady state. We did not observe changes in Flanker task and picture recall tests, which assess inhibition and memory, respectively. However, we did observe improved processing speed and global cognition, a composite score of all cognitive tests, suggesting increased blood lactate could potentially improve processing speed in both CH and CI individuals. When CH and CI groups were individually tested, we observed improved processing speed in both groups after lactate infusion, whereas global cognition was improved in the CH group and trended toward improved in the CI group. Prior work has shown that aerobic glycolysis and lactate processing could be altered in the brains of AD mouse models,^43^ which could impact the group‐specific effects we observed of lactate on global cognition. Additional work is warranted to understand how brain metabolism of lactate might be affected in AD. Improvements in cognition could be due to a learning effect, and future studies should include a saline infusion as a control to investigate this potential benefit. Studies have shown lactate to be beneficial for cognitive function, such as lactate infusion during a hyperinsulinemic‐euglycemic clamp was able to rescue cognitive impairment during hypoglycemia.^22^, ^23^ Other work has shown that lactate can still be used as a cerebral fuel source when cerebral glucose metabolism is impaired, such as in cases of traumatic brain injury (TBI), where individuals with moderate to severe TBI were able to metabolize lactate as well as control individuals despite having reduced cerebral glucose usage.^44^ Other studies have shown that lactate supplementation after TBI improves hemodynamics and metabolism, with increased cerebral pyruvate, lactate, and glucose.^45^

As an exploratory analysis, we analyzed response of AD plasma biomarkers to the lactate infusion. This resulted in one of the most notable findings in this study, which was the profound decreases in AD biomarkers such as pTau217, pTau181, BD‐Tau, NfL, and GFAP. Plasma total tau and BDNF did not change in response to LC. Because pTau217, pTau181, BD‐Tau, NfL, and GFAP are considered brain‐relevant biomarkers, we evaluated markers of BBB permeability, Claudin 5 and MMP9,^46^, ^47^ to understand if lactate could have altered clearance of these proteins from the brain. We did not observe any changes in measures of BBB permeability, suggesting this was not a mechanism underlying these effects. We found that blood volume was increased 4% by the LC, which was not related to any observed changes in AD plasma biomarkers. While pTau217 is thought to be relatively stable in concentration and has shown promising diagnostic utility,^48^ there is evidence that suggests that some AD biomarkers are acutely affected by processes such as diurnal rhythms and fasting,^30^, ^49^ which is worth noting as AB and tau have relatively long half‐lives.^50^, ^51^ Post‐translational modifications, such as tau phosphorylation or dephosphorylation, can also occur acutely; thus, these proteins may be quickly modified. It is important to recognize that changes in these plasma biomarkers acutely does not necessarily mean that the underlying disease pathology is acutely changed. However, our findings underscore the importance of understanding the underlying mechanisms of AD biomarker fluctuations in response to lactate infusion. Over time, chronic changes in mechanisms involving processes such as post‐translational modifications, lysosome activity, or other processes related to protein turnover, etc., that may be activated in an acute setting may influence disease processes.

Lactate serves as both a metabolic substrate^52^ and signaling molecule.^53^ Lactate acts as the bridge between glycolytic and oxidative metabolism, and infusion of lactate can directly support oxidative metabolism through the TCA cycle and glucose regeneration through gluconeogenesis in the liver.^15^ Lactate is a known ligand for GPR81, a G_i_‐protein coupled receptor that is thought to have roles in metabolic sensing, energy metabolism, synaptic activity, and blood flow.^53^ Our observations of altered AD biomarker expression following lactate infusion could have potentially been a result of either of these known roles of lactate. Other potential mechanisms underlying the changes in AD biomarkers may be due to physiological processes, such as liver protein catabolism or increased protein clearance through the kidneys. The liver is a primary site for the breakdown of circulating proteins and the processing of amino acids. Studies have shown that kidney function plays a critical role in plasma biomarker measures, with improved kidney function after kidney transplant leading to significant decreases in AD plasma biomarkers.^54^ To determine if the LC altered kidney function, we measured plasma creatinine to calculate the eGFR and assess changes in kidney function. In the overall cohort, there was no change in eGFR in response to lactate infusion, although when groups were compared to each other, CH had increased eGFR following infusion compared to CI individuals. However, these changes were unrelated to the observed changes in AD biomarkers.

While this study was novel due to its application of the LC in individuals with CI, it also has several limitations. This study has a relatively low sample size as it was a pilot trial aimed to investigate lactate metabolism in CI, and so the study was not powered to investigate other factors that might influence lactate metabolism, such as age, sex, and *APOE4* carrier status. The significant difference in age led us to include age adjustment (and also simultaneously for sex, including interactions between age and cognition) that we described above. These adjusted models led to similar conclusions as unadjusted results. The study did not include an isovolumetric isotonic control, which is particularly important to include for future studies to truly investigate the potential benefits of lactate on cognitive performance and biomarker fluctuations. While the NIH Toolbox has been shown to have strong test‐retest reliability when follow‐up testing is performed at 7–21 days,^55^ limited work has been done to evaluate its reliability for a 2 h follow up. Some studies have observed the administration of sodium as a counterion can confound some effects of lactate, including anorectic and thermogenic effects.^56^ Other work has shown that hypertonic sodium‐lactate infusion results in modest effects on ion, volume, and acid–base changes.^57^ However, we do not anticipate cognitive changes observed during the LC to be a result of sodium, particularly in the case of improvement of cognitive measures, as was observed in this study. The tracer lactate calculations made assumptions related to values established in young, healthy male subjects. It is possible that the volume of distribution or retention of CO_2_ could be different in the current population. In addition, while we provide the first comparison of systemic lactate metabolism, future work is needed to evaluate brain lactate metabolism, which is difficult to measure due to limited radioisotope tracer availability and the short half‐life of ^11^C.^58^ Finally, our groups were recruited based on clinical diagnosis. While AD biomarkers did differ significantly between groups, suggesting greater AD‐related neuropathology in CI participants, we did not include biomarker confirmation as part of our diagnostic characterization.

## 5 CONCLUSION

The LEAN trial characterized lactate dynamics, cognitive effects, and biomarker responses to a lactate infusion in a cohort of older adults with and without CI. We did not observe differences in whole‐body lactate metabolism in CI. This suggests that the availability of lactate produced during exercise is similar between CH and CI individuals, and that both could stand to benefit equally from lactate‐related brain benefits in response to exercise. Processing speed and global cognition improved after LC, which should be more thoroughly studied in future trials with a proper isotonic control group. We explored biomarker responses to the LC and observed profound decreases of AD‐related biomarkers. This study produced promising preliminary work that warrants further investigation of lactate metabolism in CI and its benefits to brain metabolism, cognition, and potential impact on AD biomarker levels.

## CONFLICT OF INTEREST STATEMENT

The authors declare no conflict of interest. Author disclosures are available in the Supporting Information.

## CONSENT STATEMENT

Informed consent was provided by all individuals who participated in the LEAN study.

## Supporting information



Supporting information

Supporting information

## Data Availability

Data from this study will be uploaded to Harvard Dataverse upon publication.
