# Supplementary material for: Acute effects of lactate infusion on metabolism, AD biomarkers, and cognition: The LEAN study
Source: Alzheimers Dement. 2025 Dec 10;21(12):e70984. doi: 10.1002/alz.70984 (PMC12696042; doi:10.1002/alz.70984)
Supplement: Supplementary file 1 — Supporting information [file ALZ-21-e70984-s002.docx]

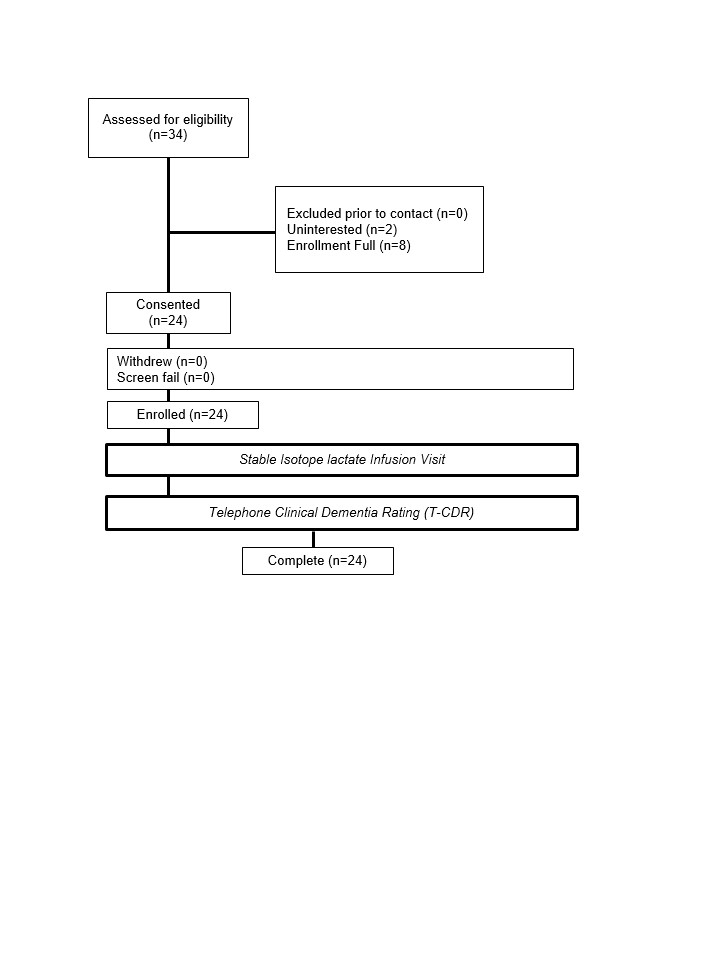


**Supplemental Figure 1. CONSORT diagram for the LEAN.** The CONSORT diagram describes participant flow through the LEAN study.


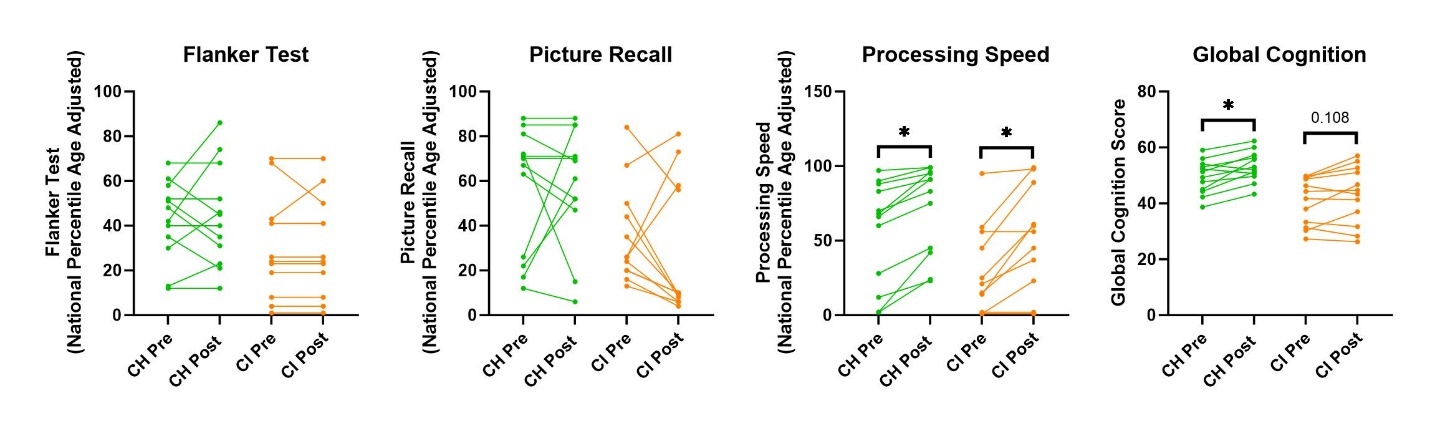


**Supplemental Figure 2. Cognitive test performance before and during lactate infusion steady state by diagnosis group.** Cognition was assessed by NIH Toolbox at baseline and after 90 minutes of lactate infusion. Results are split by cognitive diagnosis. CH; cognitively healthy, CI; cognitively impaired. *****p<0.05

**Supplemental Figure 3. Plasma pTau217 time course during lactate clamp.** Plasma pTau217 at minute 0, 10, 45, 90, and 120 in CH and AD individuals. CH; cognitively healthy, CI; cognitively impaired, pTau; phosphorylated tau.


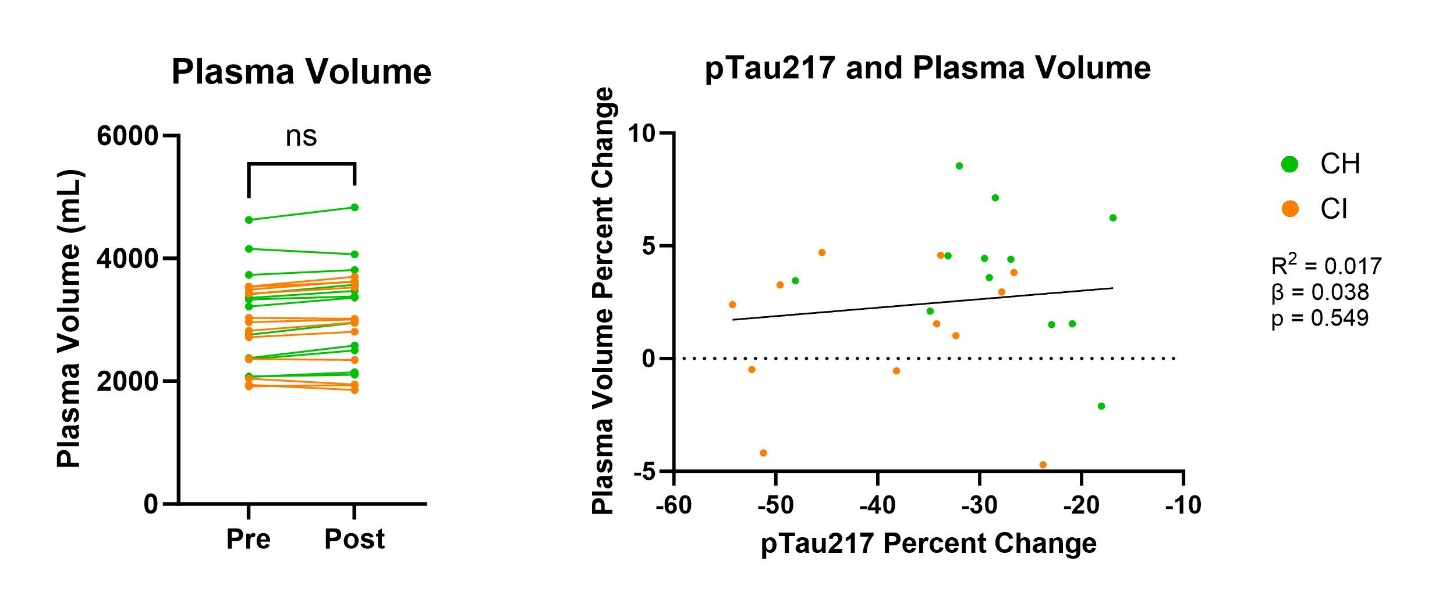


**Supplemental Figure 4. Plasma volume after lactate infusion.** Plasma volume was unchanged after lactate infusion. Plasma volume percent change and pTau217 percent change were unrelated. CH; cognitively healthy, CI; cognitively impaired; pTau; phosphorylated tau.

| Measure | **Dx** | 0 | 10 | 20 | 30 | 45 | 60 | 75 | 90 | 120 |
| --- | --- | --- | --- | --- | --- | --- | --- | --- | --- | --- |
| Hematocrit | CH | 42.5 [2.9] | 41.9 [2.9] | 40.5 [2.7] | 40.8 [2.3] | 40.1 [3.5] | 40.1 [3.1] | 39.9 [3.7] | 40.3 [3.2] | 40.3 [3.4] |
|  | CI | 42.4 [3.6] | 42.0 [3.9] | 41.4 [4.0] | 40.7 [3.8] | 41.2 [4.1] | 41.2 [4.0] | 41.2 [4.8] | 41.0 [3.5] | 41.7 [4.1] |
| Hemoglobin | CH | 14.2 [1.0] | 14.0 [1.0] | 13.5 [0.9] | 13.6 [0.7] | 13.4 [1.2] | 13.4 [1.0] | 13.3 [1.2] | 13.4 [1.1] | 13.4 [1.1] |
|  | CI | 14.1 [1.2] | 14.0 [1.3] | 13.8 [1.3] | 13.6 [1.3] | 13.7 [1.4] | 13.7 [1.3] | 13.7 [1.6] | 13.7 [1.2] | 13.9 [1.4] |
| Plasma Volume | CH | 3123.3 [818.7] | 3155.5 [830.4] | 3463.4 [1302.0] | 3445.1 [1278.9] | 3465.3 [1247.6] | 3466.7 [1258.5] | 3264.7 [873.0] | 3237.4 [817.6] | 3232.8 [813.6] |
|  | CI | 2815.9 [627.2] | 2952.5 [585.8] | 2981.2 [589.5] | 2893.3 [617.0] | 2989.1 [595.8] | 2875.9 [647.0] | 2890.5 [708.9] | 3120.7 [546.3] | 2862.8 [695.8] |

**Supplemental Table 1. Plasma volume, hematocrit, and hemoglobin throughout the lactate infusion.** Hematocrit and hemoglobin were measured at minute 0, 10, 20, 30, 45, 60, 75. 90, 120 when blood samples were collected. Plasma volume was calculated for each timepoint using hematocrit measures.
